# Bendiocarb resistance in *Anopheles gambiae s.l*. populations from Atacora department in Benin, West Africa: a threat for malaria vector control

**DOI:** 10.1186/1756-3305-6-192

**Published:** 2013-06-26

**Authors:** Rock Aïkpon, Fiacre Agossa, Razaki Ossè, Olivier Oussou, Nazaire Aïzoun, Frédéric Oké-Agbo, Martin Akogbéto

**Affiliations:** 1 Centre de Recherche Entomologique de Cotonou (CREC), 06 BP 2604, Cotonou, Bénin; 2 Faculté des Sciences et Techniques, Université d’Abomey Calavi, Calavi, Bénin

**Keywords:** Bendiocarb resistance, *Anopheles gambiae*, Threat, Malaria vector control, Benin

## Abstract

**Background:**

Owing to pyrethroid resistance in *An*. *gambiae*, the carbamate and organophosphate insecticides are currently regarded as alternatives or supplements to pyrethroids for use on mosquito net treatments. Resistance monitoring is therefore essential to investigate the susceptibility of *An*. *gambiae s*.*l* to these alternative products.

**Methods:**

Two to three day old adult female Anopheles mosquitoes were reared from larvae collected in the five districts (Kouandé, Natitingou, Matéri, Péhunco, Tanguiéta) of the Atacora department. Mosquitoes were then exposed to WHO impregnated papers. The four treatments consisted of: carbamates (0.1% bendiocarb, 0.1% propoxur) and organophosphates (0.25% pirimiphosmethyl, 1% fenitrothion). PCR assays were run to determine the members of the *An*. *gambiae* complex, the molecular forms (M) and (S), as well as phenotypes for insensitive acetylcholinesterase (AChE1) due to *ace*-*1*^*R*^ mutation.

**Results:**

Bioassays showed bendiocarb resistance in all populations of *An*. *gambiae s*.*s*. tested. Propoxur resistance was observed in Matéri, Péhunco and Tanguiéta, while it was suspected in Kouandé and Natitingou. As for the organophosphates, susceptibility to pirimiphos-methyl was assessed in all populations. Fenitrothion resistance was detected in Kouandé, Péhunco and Tanguiéta, while it was suspected in Matéri and Natitingou. The S-form was predominant in tested samples (94.44%). M and S molecular forms were sympatric but no M/S hybrids were detected. The *ace*-*1*^*R*^ mutation was found in both S and M molecular forms with frequency from 3.6 to 12%. Although the homozygous resistant genotype was the most prevalent genotype among survivors, the genotypes could not entirely explain the bioassay results.

**Conclusion:**

Evidence of bendiocarb resistance in *An*. *gambiae* populations is a clear indication that calls for the implementation of insecticide resistance management strategies. The *ace*-*1*^*R*^ mutation could not entirely explain the resistance to bendiocarb observed and is highly suggestive of involvement of other resistance mechanisms such as metabolic detoxification.

## Background

Malaria is a major public health problem and *Anopheles gambiae* is one of the major vectors of this disease in sub-Saharan Africa [1]. The current effective vector control tools include the use of Long Lasting Insecticide Nets (LLIN) and Indoor Residual Spraying (IRS) [2]. In sub-Sahara Africa and southern Asia, these two methods have shown good results [3,4].

Pyrethroids are the only group of insecticides currently recommended for net treatment, the others (organochlorine, carbamate and organophosphate) are applied for IRS [5,6]. The main problem with ITNs and IRS is the development of insecticide resistance, particularly pyrethroid-resistance by several populations of *Anopheles gambiae*[7-10]. Prior to the present study, a monitoring survey was carried out on pyrethroid resistance from January to October 2012 in the department of Atacora and showed a high level of kdr allelic frequency of 81.78% on average. The kdr mutation was found in both S (92.02%) and M (30.25%) molecular forms (Aïkpon, personal communication). More recently, the emergence of resistance in populations of *An*. *gambiae* to common classes of insecticides used in public health has been reported in many countries in Africa, including Côte d’Ivoire [7,11], Kenya [12], Benin [13,14], Niger [15], Burkina Faso [16], Mali [17], Nigeria [18,19], South Africa [20] and Cameroon [21].

With the widespread resistance to pyrethroids, the carbamate class of insecticides is one of the possible alternatives that can be considered effective enough to combat pyrethroid-DDT resistance, mainly because of its different mode of action. For this reason, Benin Republic adopted a national malaria control strategy based on large-scale integrated control measures, which included Insecticide Treated Nets (ITNs) and Indoor Residual Spraying (IRS) using bendiocarb, a carbamate insecticide. The department of Atacora has housed a large scale IRS campaign since 2011. However, there is not sufficient information on the resistance status of carbamate insecticides in the field populations of *An*. *gambiae s*.*l*. in North West Benin.

The aim of this study is to provide information on the susceptibility status of *An*. *gambiae s*.*l*. to carbamate that has been used in vector control in Benin and also to investigate the possibility of co-resistance with organophosphates in the same population of *An*. *gambiae s*.*l*.. It is hoped that findings from this study will promote and improve effective vector control decision making.

## Methods

### Study area

**Figure 1:**
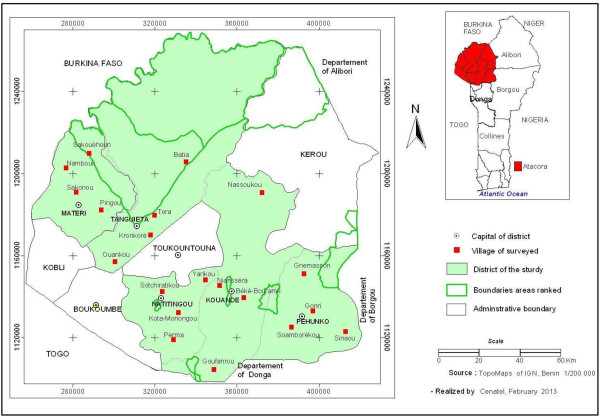
Map of the department of Atacora showing the localities where Anopheline mosquitoes were collected.

The study was carried out in Atacora, a department located in the north-west of Benin and includes five districts: Kouandé, Matéri, Natitingou, Péhunco and Tanguiéta (Figure 1). The five districts covered 13,778 km^2^ and an estimated 482,080 populations in 2012. Atacora region has a sub-equatorial type climate with only one dry season (December-May) and only one rainy season (July- November). The annual mean rainfall is 1,300 mm and the mean monthly temperature varies between 22°C and 33°C. The department is irrigated by three major rivers: the Mekrou, the Pendjari and the Alibori. The major economic activity is agriculture and it is characterized by the production of cotton and millet where various classes of pesticides are used for pest control. From 2011 onwards the department has conducted a large scale Indoor Residual Spraying (IRS) campaign and free distribution of ITNs.

### Mosquito collections

*Anopheles gambiae s*.*l*. larvae were collected in 5 districts and in each district, four villages were selected randomly. At each locality chosen, Anopheline larvae were collected from various natural breeding sites including ground pools, gutters, puddles and abandoned potholes, during the rainy season from July to October 2012. Water was scooped using a plastic scoop and poured into small transparent plastic bowls. A strainer was used to sieve and pool together the third and fourth instar larvae in order to have sufficient adult emergence of the same physiological age. The mosquito larvae collected were transported in well labeled plastic bottles to the laboratory of the Centre de Recherche Entomologique de Cotonou, Benin (CREC) where they were maintained at 28 ± 2 C and 72 ± 5% relative humidity. A laboratory susceptible strain of *An*. *gambiae* Kisumu was used as a reference strain to compare the susceptibility levels of the field populations.

### Insecticide susceptibility tests

Mosquitoes collected were assayed using WHO discriminating dosages with four insecticides of technical grade quality: two carbamates (0.1% bendiocarb, 0.1% propoxur) and two organophosphates (0.25% pirimiphos méthyl, 1% fenitrothion). Four batches of 25 unfed females, aged 2–5 days, were exposed to the diagnostic doses of insecticide treated papers for 60 min at 27 ± 1°C and 80% relative humidity. The twenty-five females of *An*. *gambiae* were introduced into each tube and monitored at different time intervals (10, 15, 20, 30, 45, 60 minutes), the number “knocked-down” were recorded. After one hour exposure, mosquitoes were transferred into holding tubes and provided with cotton wool saturated with a 10% honey solution. Batches exposed to untreated papers were used as control. Mortalities were recorded after 24 hours and the susceptibility status of the population was graded according to the WHO protocol [22]. Dead and surviving mosquitoes from this bioassay were kept separately in eppendorf tubes containing silica gel and stored at −20°C for further molecular analysis.

### Species identification and PCR detection of *Ace*-*1*^*R*^ mutation

Live and dead specimens of *An*. *gambiae* from the bioassay tests were subjected to the *An*. *gambiae* species specific PCR assays for species identification [23]. Aliquots of DNA extracted from PCR positive specimens of *An*. *gambiae s*.*s*. were subjected to PCR assays for identification of the molecular ‘M’ and ‘S’ forms [24].

The PCR-RFLP diagnostic test was used to detect the presence of G119S mutation (*ace*.*1*^*R*^ gene). Mosquito genomic DNA was amplified using the primers Ex3AGdir 5′GATCGTGGACACCGTGTTCG3′ and Ex3AGrev 5′AGGATGGCCCGCTGGAACAG3′ according to [25]. One microlitre of total DNA extracted from a single mosquito was used as a template in a 25 ml PCR reaction containing *Taq* DNA polymerase buffer, 0.2 mM dNTP and 10 pmol of each primer. The PCR conditions were 94°C for 5min and then 35 cycles of (94°C for 30 s, 54°C for 30 s and 72°C for 30 s) with a final 5 min extension at 72°C. Fifteen microlitres of PCR product were digested with 5U of AluI restriction enzyme (Promega) in a final volume of 25ml. The PCR fragments were fractionated on a 2% agarose gel stained with ethidium bromide and visualized under UV light.

### Data analysis

The resistant status of mosquito samples was determined according to the WHO criteria [22]:

• Mortality rate is > 98%: the population was considered fully susceptible

• Mortality rates ranged between 90 - 98%: resistance suspected in the population

• Mortality rates < 90%, the population was considered resistant to the tested insecticides

To compare the status of insecticide resistance, Fisher's exact test was carried out to determine if there was any significant difference between mortality rates of populations of *An*. *gambiae s*.*s*. of districts using Statistica 6.0. Allelic frequencies of G119S mutation were analysed using the version 1.2 of Genepop [26]. To assess if the mutation frequencies were identical across populations, the test of genotypic differentiation was performed [27].

## Results

### Susceptibility to carbamates and organophosphates

Mortality rates of the Kisumu reference strain to all insecticides was 100% (Table 1). In contrast, all the field samples (Kouandé, Matéri, Natitingou, Péhunco and Tanguiéta) were resistant to carbamates, with mortality rates less than 80% for bendiocarb. Propoxur resistance was observed in Matéri, Péhunco and Tanguiéta, with 79-89% mortality rates, while it was suspected in Kouandé and Natitingou with 90-91% mortality rate. As for the organophosphates, susceptibility to pirimiphos-methyl was assessed on all populations with mortality rates higher than 98%. Fenitrothion resistance was detected in Kouandé, Péhunco and Tanguiéta, with 83-90% mortality rates, while it was suspected in Matéri and Natitingou with 94-95% mortality rates.

**Table 1:** **Mortality of a susceptible strain (Kisumu) and wild populations of *Anopheles gambiae s.s. *exposed to diagnostic doses of technical material of insecticides**

**Localities**	**Bendiocarb 0**.**1%**		**Propoxur 0.1%**		**Pirimiphos-méthyl 0.25%**		**Fenitrothion 1%**	
	**% Mortality**	**Status**	**% Mortality**	**Status**	**% Mortality**	**Status**	**% Mortality**	**Status**
Kisumu	100(103)	S	100 (102)	S	100 (99)	S	100 (104)	S
Kouandé	78.89^a^ (90)	R	90^a^ (60)	RS	100^a^ (81)	S	87.8^a^ (82)	R
Matéri	58.9^b^ (73)	R	89^a^ (70)	R	100^a^ (72)	S	94.79^a^ (96)	RS
Natitingou	61.9^b^ (84)	R	91^a^ (62)	RS	100^a^ (58)	S	95^a^ (70)	RS
Péhunco	79.21^a^ (101)	R	79.69^a^ (64)	R	98.88^a^ (89)	S	89.89^a^ (89)	R
Tanguiéta	63.21^b^ (106)	R	88.13^a^ (59)	R	100^a^ (90)	S	83.67^a^ (49)	R

Number of tested mosquitoes in parentheses; % Mortality: Mortality rate 24 h post exposure; S: indicates susceptibility; R: suggests resistance; RS: resistance suspected.

NB: Numbers in the same column with the same superscript do not differ significantly by Fisher's exact test (p > 0.05).

### Molecular forms and frequencies of the *ace*-*1*^*R*^ mutation

All PCR analysis identifying *An*. *gambiae s*.*l*. species realized in this study showed that all mosquitoes belonging to *An*. *gambiae s*.*l*. were *An*. *gambiae s*.*s*. Two hundred and fifty-two mosquitoes were identified to molecular forms and analyzed for the *ace*.*1*^*R*^ mutation; results are shown in Table 2. The M and S molecular forms of *An*. *gambiae* s.s. occurred in sympatry in Kouandé, Matéri, Natitingou and Tanguiéta districts. However, the S-form was predominant, representing 94.44% of the whole sample (n=252). No heterozygote M/S molecular form was found. The *ace*-*1*^*R*^ mutation was detected in all the districts, either in the M or in the S form. It was detected both in the homozygous and heterozygote state in S form, but only in the heterozygote state in M form with only one individual mutant. So, the highest mutation frequency was observed in the S form (12%) and the lowest in the M (3.6%). No significant difference was seen between *ace*.*1*^*R*^ mutation frequencies in the districts in addition, no significant difference was seen between *ace*.*1*^*R*^ mutation frequencies in M and S forms (p = 0.2327).

**Table 2:** **Acetylcholinesterase phenotypes and frequency of *ace-1***^***R ***^**mutation in the molecular M and S forms of *Anopheles gambiae s.s***

**Localities**	**S Form**				**M Form**			
	**Phénotypes**			**f( *ace *- *1 *)**	**Phénotypes**			**f( *ace *- *1 *)**
	**RR**	**RS**	**SS**		**RR**	**RS**	**SS**	
Kouandé	1	10	47	0,103^a^	0	0	3	0
Matéri	2	6	36	0,114^a^	0	0	4	0
Natitingou	0	11	33	0,125^a^	0	0	1	0
Péhunco	1	11	37	0,133^a^	0	0	0	-
Tanguiéta	0	11	32	0,128^a^	0	1	5	0,083
**Total**	4	49	185	0,12	0	1	13	0,036

^a^Values sharing a superscript letter are not significantly different at the 5% level for G119S mutation distribution.

### Role of *ace*-*1*^*R*^ mutation in providing bendiocarb resistance

**Figure 2:**
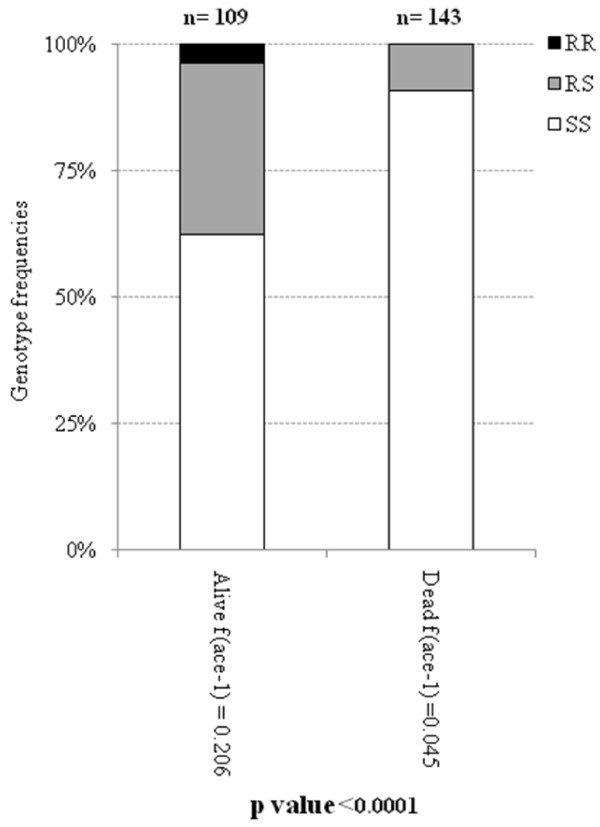
***ace-1***^***R ***^**genotypes frequencies found in live and dead *An. gambiae s.s *individuals from WHO susceptibility test to bendiocarb.**

To assess the role of the *ace*-*1*^*R*^ allele in conferring bendiocarb resistance in *An*. *gambiae s*.*s*., the *ace*-*1*^*R*^ genotype was determined for dead and alive mosquitoes detected in the WHO bioassay using bendiocarb (Figure 2). Among both bioassay survivors and non-survivors, all *ace*-*1*^*R*^ genotypes (RR, RS and SS) were found. However, the homozygous resistant genotype RR was only found among the bioassay survivors, and the heterozygote genotype RS was the most prevalent genotype among the bioassay survivors.

Although there was a significant *ace*-*1*^*R*^ genotype differentiation between bioassay survivors and non-survivors (p<0.0001), homozygous susceptible mosquitoes were found among bioassay survivors.

## Discussion

Information on the resistance status of the main malaria vectors is essential to guide the choice of insecticides for use by The National Malaria Control Programme. Indeed, since 2011, the NMCP of Benin has implemented a large IRS campaign using bendiocarb in the department of Atacora. There is,therefore, the need to closely monitor insecticide resistance and bendiocarb resistance especially in malaria control programmes which rely solely on ITNs and IRS interventions in Benin. Moreover, very little data is available on the status of *An*. *gambiae* resistance to carbamates and organophosphates in the department of Atacora.

Results of this study showed that field populations of *An*. *gambiae* collected from five districts of the department of Atacora developed resistance to bendiocarb, propoxur, and fenitrothion but not to pirimiphos-methyl. This is the first time that bendiocarb resistance has been reported in Benin. Indeed, previous studies in the department of Atacora reported that *An*. *gambiae* were susceptible to bendiocarb in 2010 and justified its choice in IRS in this department (Aïkpon, personal communication). Akogbeto *et al*. [28] and Padonou *et al*. [29] reported this susceptibility of *An*. *gambiae* to bendiocarb in southern Benin. Moreover, *An*. *gambiae* displayed large variations in resistance levels to carbamates and organophosphates. Although the wild populations were all resistant to bendiocarb, resistance was less marked to propoxur and fenitrothion, at WHO diagnostic concentrations. However, all these populations were very susceptible to pirimiphos-methyl. The high resistance of the mosquito population to bendiocarb would be due to the strong selective pressure that represents the use of insecticides in households for public health purposes, notably IRS using bendiocarb and massive quantities of carbamates and organophosphates in agricultural settings in the department of Atacora. Indeed, in the cotton growing areas in Atacora, farmers use huge amounts of insecticides to avoid substantial yield reduction of their crops. Several studies showed that agricultural practices seem to have contributed to the emergence of insecticide resistance in *Anopheles* populations [10,14,30].

The development of resistance by the mosquito population to bendiocarb could jeopardize the current malaria control programme, specifically IRS using bendiocarb that is currently underway in the department of Atacora.

Cross-resistance to organophosphates and carbamates suggests the involvement of their common target site: AChE-1 [31]. Indeed, the *ace*-*1*^*R*^ mutation was identified in all districts although its frequency remains relatively low, and agrees with previous findings that reported *ace*-*1*^*R*^ mutation in Benin [32].

In this study, the distribution of M and S molecular forms of *An*. *gambiae* s.s. agrees with previous findings in Benin that reported both M and S forms with the predominance of S forms in a savannah areas [33]. The presence of *ace*-*1*^*R*^ mutations in both M and S forms of *An*. *gambiae s*.*s*. has already been reported by Weill *et al*. [31] and Djogbénou *et al*. [32] and was suggested to result from introgression between forms. However, the *ace*-*1*^*R*^ mutation frequency was higher in the S form. The low number of homozygous resistant individuals might be related to high fitness cost of the *ace*-*1*^*R*^ mutation, resulting in death of the homozygous resistant mosquitoes [31,34,35]. The high number of heterozygous resistant RS is also in agreement with previous studies that noticed that in areas where the resistant allele *ace*-*1*^*R*^ is present, resistant mosquitoes will mainly be in the heterozygote state (RS) [11,35].

Moreover, the role of *ace*-*1*^*R*^ mutation in conferring bendiocarb resistance was assessed. The WHO bioassays performed on *An*. *gambiae s*.*s*. from the study area showed that the homozygous resistance was found only among bioassay survivors, however, the homozygous susceptible genotype (SS) is the most prevalent genotype among these survivors. The high proportion of homoygous susceptible specimens, which survived the WHO bioassays, added to the low rate of *ace*-*1*^*R*^ allele frequency may suggest the implication of biochemical resistance mechanisms.

Further investigation is needed to evaluate the biochemical mechanism that could be involved in the resistance of *An*. *gambiae* to carbamates and organophosphates and better understand the difference in resistance between the carbamates and organophosphates.

## Conclusion

The present study provides useful information on the susceptibility of *An*. *gambiae* to carbamates and organophosphates. It showed that *An*. *gambiae* has developped a resistance to bendiocarb that can be a threat for malaria vector control in Benin. Hence, there is a need to implement vector resistance management approches to malaria vector control in Benin.

## Competing interests

The authors declare that they have no competing interests.

## Author’s contributions

RA, FA, RO, OO, NA, FOA and MA designed the study. RA, OO, NA and MA carried out the field activities. RA drafted the manuscript and analyzed the data. FA, RO and FOA critically revised the manuscript. MA conceived and designed the study and revised the manuscript for intellectual content. All authors read and approved the final manuscript.
